# Reasons for hospitalisation and cumulative mortality in people, 75 years or older, at high risk of hospital admission: a prospective study

**DOI:** 10.1186/s12877-024-04771-2

**Published:** 2024-02-20

**Authors:** Moa Lundgren, Anna Segernäs, Magnus Nord, Jenny Alwin, Johan Lyth

**Affiliations:** 1 Primary Health Care Centre Finspång, Finspång, Sweden; 2 Department of Health, Medicine and Caring Sciences, Linköping University, Linköping, Sweden; 3 Primary Health Care Centre Ekholmen, Linköping, Sweden; 4 Primary Health Care Centre Valla, Linköping, Sweden

**Keywords:** Aged, Older people, Prediction model, Hospitalisation, Hospital admission, Mortality, Frailty

## Abstract

**Background:**

A small proportion of the older population accounts for a high proportion of healthcare use. For effective use of limited healthcare resources, it is important to identify the group with greatest needs. The aim of this study was to explore frequency and reason for hospitalisation and cumulative mortality, in an older population at predicted high risk of hospital admission, and to assess if a prediction model can be used to identify individuals with the greatest healthcare needs. Furthermore, discharge diagnoses were explored to investigate if they can be used as basis for specific interventions in the high-risk group.

**Methods:**

All residents, 75 years or older, living in Östergötland, Sweden, on January 1^st^, 2017, were included. Healthcare data from 2016 was gathered and used by a validated prediction model to create risk scores for hospital admission. The population was then divided into groups by percentiles of risk. Using healthcare data from 2017–2018, two-year cumulative incidence of hospitalisation was analysed using Gray´s test. Cumulative mortality was analysed with the Kaplan–Meier method and primary discharge diagnoses were analysed with standardised residuals.

**Results:**

Forty thousand six hundred eighteen individuals were identified (mean age 82 years, 57.8% women). The cumulative incidence of hospitalisation increased with increasing risk of hospital admission (24% for percentiles < 60 to 66% for percentiles 95–100). The cumulative mortality also increased with increasing risk (7% for percentiles < 60 to 43% for percentiles 95–100). The most frequent primary discharge diagnoses for the population were heart diseases, respiratory infections, and hip injuries. The incidence was significantly higher for heart diseases and respiratory infections and significantly lower for hip injuries, for the population with the highest risk of hospital admission (percentiles 85–100).

**Conclusions:**

Individuals 75 years or older, with high risk of hospital admission, were demonstrated to have considerable higher cumulative mortality as well as incidence of hospitalisation. The results support the use of the prediction model to direct resources towards individuals with highest risk scores, and thus, likely the greatest care needs. There were only small differences in discharge diagnoses between the risk groups, indicating that interventions to reduce hospitalisations should be personalised.

**Trial registration:**

clinicaltrials.gov Identifier: NCT03180606, first posted 08/06/2017.

## Background

A small proportion of the older population accounts for a high proportion of healthcare use [1–4]. For effective use of limited healthcare resources, it is of great importance to identify the group with the greatest needs. Individuals with multimorbidity and/or frailty are more likely to be admitted to hospital [5, 6]. In addition, frail individuals have a higher mortality rate than others [7]. The prevalence of both multimorbidity and frailty increases with age [8–10]. People 65 years or older have a hospitalisation prevalence of 5.6 to 25.8 percent over 12 months [11]. Data from previous studies describing diagnostic reasons for hospitalisation in older people is scarce. Older people seem, however, to be hospitalised most frequently due to diseases in the circulatory and respiratory system [12, 13]. Men seem to be hospitalised more frequently due to cancer and diseases in the respiratory system than women, while women seem to be hospitalised more frequently due to fractures or falls than men [12].

In Sweden, primary care consists of more than 1 000 health centres and clinics, which are the first line of contact to the healthcare system for the whole population. These health centres are responsible for cooperating with other levels of care as well as the municipalities, which are responsible for long term care such as nursing homes and domestics services [14].

The world is facing a situation with an increasing number of older people [15, 16]. Hence, it is important to have methods to direct the resources to the individuals with the highest risk of hospital admission when planning healthcare interventions preventing decline in health [17, 18]. Different ways of predicting patients with high healthcare use have been suggested, including frailty [7, 19, 20] and multimorbidity [6, 10]. Many strategies for predicting high-risk patients rely on clinical assessments or self-administrated questionnaires. These screening procedures are costly, and some patients at risk will go unnoticed due to lower response rate in groups with characteristics associated with risk of high healthcare use [21]. The use of healthcare databases to predict who will be admitted to hospital has been suggested to be a more feasible approach [22–28]. To focus on prevention of hospitalisation is not only relevant from a cost perspective; hospitalisation is also associated with higher risk of admission to nursing home [29] and functional decline, which negatively impacts the quality of life of the individual [30].

To identify individuals with a probable benefit from interventional programs, Marcusson et al. [31] designed a model aiming to predict hospital admission among people, 75 years or older, in the coming twelve months. The prediction model was created to be used in a pragmatic intervention trial in primary care, called *Proactive healthcare for frail elderly persons*. In this trial, individuals identified by the model to have a high risk of hospital admission, were assessed with comprehensive geriatric assessment (CGA) adapted to primary care [32]. The aim was to investigate if personalised, targeted primary care could reduce hospitalisations compared with usual care. The assessment of identified people, 75 years or older, resulted in a 22 percent relative risk reduction for inpatient-hospital days [33]. The intervention was cost-effective [34].

The knowledge about primary discharge diagnoses and how they differ between individuals at different levels of risk of hospitalisation is scarce. An identifiable difference in distribution of diagnoses could form the basis for targeted intervention efforts in different risk groups of frail old people. This can be of value, for example in primary care, in efforts to organise cost-effective care for large populations and to proactively reach older individuals at high risk of morbidity, hospitalisation, and increased risk of mortality.

The present study examines how the cumulative incidence of hospitalisation, the reason for hospitalisation, and the cumulative mortality varies at different levels of risk of hospital admission, in a Swedish population at an age of 75 years or older, to further validate the prediction model developed by Marcusson et al. [31].

### Aim

The aim of this study was to explore frequency and reason for hospitalisation and cumulative mortality, in an older population at predicted high risk of hospital admission and to assess if a prediction model can be used to identify individuals with the greatest healthcare needs. The aim was further to examine if differences in distribution of primary and secondary discharge diagnoses could form the basis for targeted intervention efforts according to gender, or to risk groups of older people.

## Methods

### Setting and population

The study is a prospective registry-based cohort study that included all residents, 75 years or older on January 1st, 2017, in the county of Östergötland in the southeast part of Sweden. Healthcare data consisting of gender, age, and diagnoses from both hospital care and open clinic visits (grouped by two digits) according to International Classification of Diseases (ICD-10) [35] from the year of 2016, was gathered from the Care Data Warehouse (CDW) of Region Östergötland. The CDW is a computerised information system where healthcare use for the region is stored. The number of hospital admissions (at the latest initiated on December 31st, 2016) and the number of non-physician (for example nurses, occupational therapists, or physiotherapists), physician, and emergency room (ER) visits were also included. In total, 37 variables were used in a prediction model to create a risk score for hospital admission for each person [31]. The number of hospital admissions (initiated between January 1st, 2017, and December 31st, 2018), the related primary and secondary discharge diagnoses, and the mortality during 2017 and 2018 were also collected from the CDW. If an individual died during hospitalisation this was regarded both as a hospital admission episode and as a death. In those cases, the first registered diagnosis was regarded as the discharge diagnosis.

The study population was divided into risk groups by percentiles of risk, derived from the predicted risk of hospital admission. Individuals with risk percentiles < 60 were regarded as one group as these individuals were considered less clinically relevant when primarily screening for high-risk patients, which is the main purpose of the prediction model [31]. This group was therefore not further analysed. Individuals with risk percentiles 60 to 100 was divided in groups of five percentile points.

### Statistics

The cumulative incidence of hospitalisation in the different risk groups was analysed using Gray´s test. The subdistribution hazard ratios (SHR) were analysed with Fine-Gray subdistribution hazard model, treating mortality as competing risk [36, 37]. The cumulative mortality rates were calculated with the Kaplan–Meier method, and the hazard ratios were analysed with Cox-regression. The analyses were also conducted according to gender, and confidence intervals were compared to find any significant difference between gender.

Each individual’s primary discharge diagnosis of their first episode of hospitalisation were analysed, both as blocks, according to ICD-10 [38], and as ICD-10 diagnoses (grouped by two digits). ICD-10 blocks were sorted by the most frequent to the least frequent. ICD-10 blocks accounting for at least two percent of the primary discharge diagnoses for the population in total were analysed separately. The remaining ICD-10 blocks were clustered into one group called *other diagnoses*. Based on the results from the analysis on hospitalisation, where the cumulative incidence overlapped up to percentile 85, the ICD-10 blocks were analysed in five groups: percentiles < 60, 60–85, 85–90, 90–95, and 95–100 and in three groups: percentiles < 60, 60–85, and 85–100. The distribution of the top ten ICD-10 diagnoses for the entire population were presented in total and according to gender. The primary discharge blocks and diagnoses were analysed with Chi-2 and standardised residuals (Pearson residuals) to find significant differences between risk groups and gender. Each individual´s secondary discharge diagnoses (ICD-10 diagnoses grouped by two digits) of their first episode of hospitalisation were analysed with Chi-2 and standardised residuals. Top five secondary discharge diagnoses were presented. Statistical analyses were performed in R v.4.2.1. Gray and Fine-Gray analyses were computed in the cmprsk-package.

## Results

In total, 40 618 individuals, 75 years or older, living in Östergötland, Sweden, on January 1st, 2017, were identified. The background characteristics of the population are reported in Table 1. Mean age was 82 years, and 57.8 percent of the population were women. The percentage of women decreased with increasing risk for hospitalisation in the groups.

**Table 1:** Characteristics of the study population at baseline and their healthcare use during 2016

Percentiles of risk of hospital admission	Number of individuals	Age, mean (SD)	Women, %	Number of physician visits, mean (SD)	Number of non-physician visits, mean (SD)	Number of visits at the ER, mean (SD)	Number of hospital admissions, mean (SD)
0–60	24 386	79.9 (4.1)	60.2	2.3 (2.5)	4.9 (5.8)	0.1 (0.4)	0.1 (0.3)
60–65	2 017	84.7 (5.5)	56.9	3.7 (3.3)	7.9 (8.6)	0.3 (0.6)	0.2 (0.5)
65–70	2 030	85.1 (5.8)	59.2	4.2 (3.9)	8.3 (8.9)	0.4 (0.7)	0.2 (0.5)
70–75	2 036	85.3 (6.1)	55.9	4.5 (4.3)	8.9 (10.1)	0.5 (0.7)	0.3 (0.6)
75–80	2 037	85.2 (6.1)	55.6	5.1 (4.7)	10.6 (11.0)	0.6 (0.9)	0.4 (0.7)
80–85	2 033	85.3 (6.0)	54.9	5.6 (4.6)	10.7 (11.6)	0.8 (0.9)	0.5 (0.7)
85–90	2 019	85.4 (6.1)	53.0	6.6 (5.3)	13.0 (14.4)	1.0 (1.1)	0.7 (0.8)
90–95	2 031	85.5 (6.0)	52.0	7.8 (5.4)	15.8 (18.8)	1.4 (1.3)	1.0 (1.0)
95–100	2 029	84.7 (5.8)	45.5	11.7 (7.8)	23.4 (27.7)	2.7 (2.5)	1.9 (1.6)
Total	40 618	82.0 (5.5)	57.8	3.8 (4.5)	7.8 (11.6)	0.5 (1.0)	0.3 (0.7)

The total median values (interquartile range, min–max) for each variable were: age = 81 (9, 75–108), physician visits = 3 (4, 0–103), non-physician visits = 4 (8, 0–327), visits at the ER = 0 (1, 0–36), hospital admissions = 0 (1, 0–13)

*Abbreviations: SD* standard deviation, *ER* emergency room

**Fig. 1:**
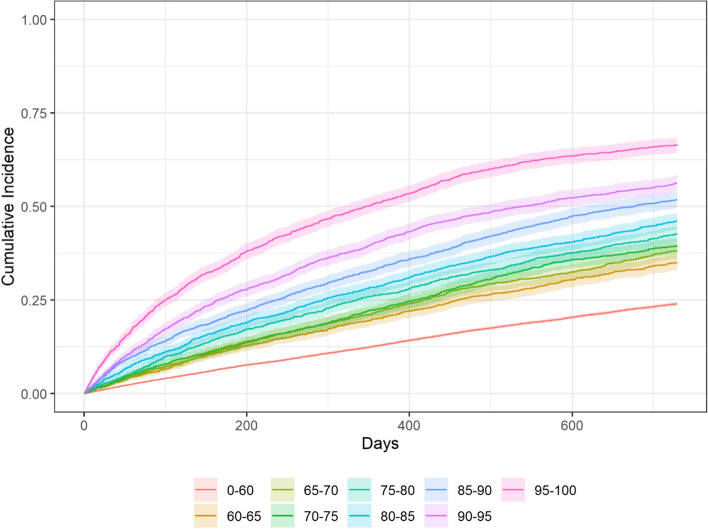
2-year cumulative incidence of hospitalisation by percentiles of risk of hospital admission, year 2017–2018. Estimated using Gray´s test

In total, 8 131 individuals had experienced at least one episode of hospitalisation after one year and 13 491 after two years. The individuals in the higher risk groups had higher cumulative incidence of hospitalisation. The cumulative incidence, during a follow-up period of two years, varied between 24 percent in the lowest (percentiles < 60) compared to 66 percent in the highest risk group (percentiles 95–100). The confidence intervals overlapped between adjacent risk groups from percentiles 60–65 up to percentiles 80–85 and were thereafter separated between the highest risk groups (percentiles 85–90, 90–95, and 95–100). There was no significant difference in hospitalisation between men and women when divided into risk groups. Data on cumulative incidence and SHR are presented in Table 2 and the cumulative incidence is also presented in Fig. 1.

**Table 2:** Number of hospital admissions, 2-year cumulative incidence of hospitalisation and subdistribution hazard ratios, year 2017–2018

Percentiles of risk of hospital admission	Number of individuals	Number of individuals with ≥ 1 hospital admissions	2-year cumulative incidence^a^, % (95% CI)	Subdistribution hazard ratios^b ^(95% CI)	Total number of hospital admissions	Number of hospital admissions, mean (SD)	Number of inpatient-hospital days, mean (SD)
0–60	24 386	5 858	24 (23–25)	-	8 868	0.4 (0.8)	2.2 (6.4)
60–65	2 017	708	35 (33–37)	1.6 (1.5–1.7)	1 154	0.6 (1.0)	3.5 (7.8)
65–70	2 030	776	38 (36–40)	1.8 (1.6–1.9)	1 281	0.6 (1.0)	3.9 (7.8)
70–75	2 036	805	40 (37–42)	1.8 (1.7–2.0)	1 334	0.7 (1.1)	4.1 (8.2)
75–80	2 037	870	43 (41–45)	2.0 (1.9–2.2)	1 446	0.7 (1.1)	4.3 (8.2)
80–85	2 033	938	46 (44–48)	2.3 (2.1–2.4)	1 764	0.9 (1.3)	5.4 (9.8)
85–90	2 019	1 047	52 (50–54)	2.7 (2.6–2.9)	1 979	1.0 (1.4)	6.2 (10.5)
90–95	2 031	1 141	56 (54–58)	3.2 (3.0–3.4)	2 261	1.1 (1.4)	7.1 (11.2)
95–100	2 029	1 348	66 (64–68)	4.1 (3.8–4.4)	2 457	1.7 (2.1)	10.6 (15.3)
Total	40 618	13 491	33 (33–34)	-	23 489	0.6 (1.1)	3.6 (8.4)

Presented by percentiles of risk of hospital admission

*Abbreviations*: *CI* confidence interval

^a^Estimated using Gray´s test accounting for mortality as competing risk

^b^Estimated using Fine-Gray subdistribution hazard model accounting for mortality as competing risk

With a follow-up time of two years, the mean inpatient-hospital days were 3.6 days, ranging from 2.2 days in the lowest risk group (percentiles < 60) to 10.6 days in the highest risk group (percentiles 95–100). The mean number of days during the first hospitalisation episode was 6.2, ranging from 6.0 (percentiles < 60) to 6.4 (percentiles 95–100).

**Fig. 2:**
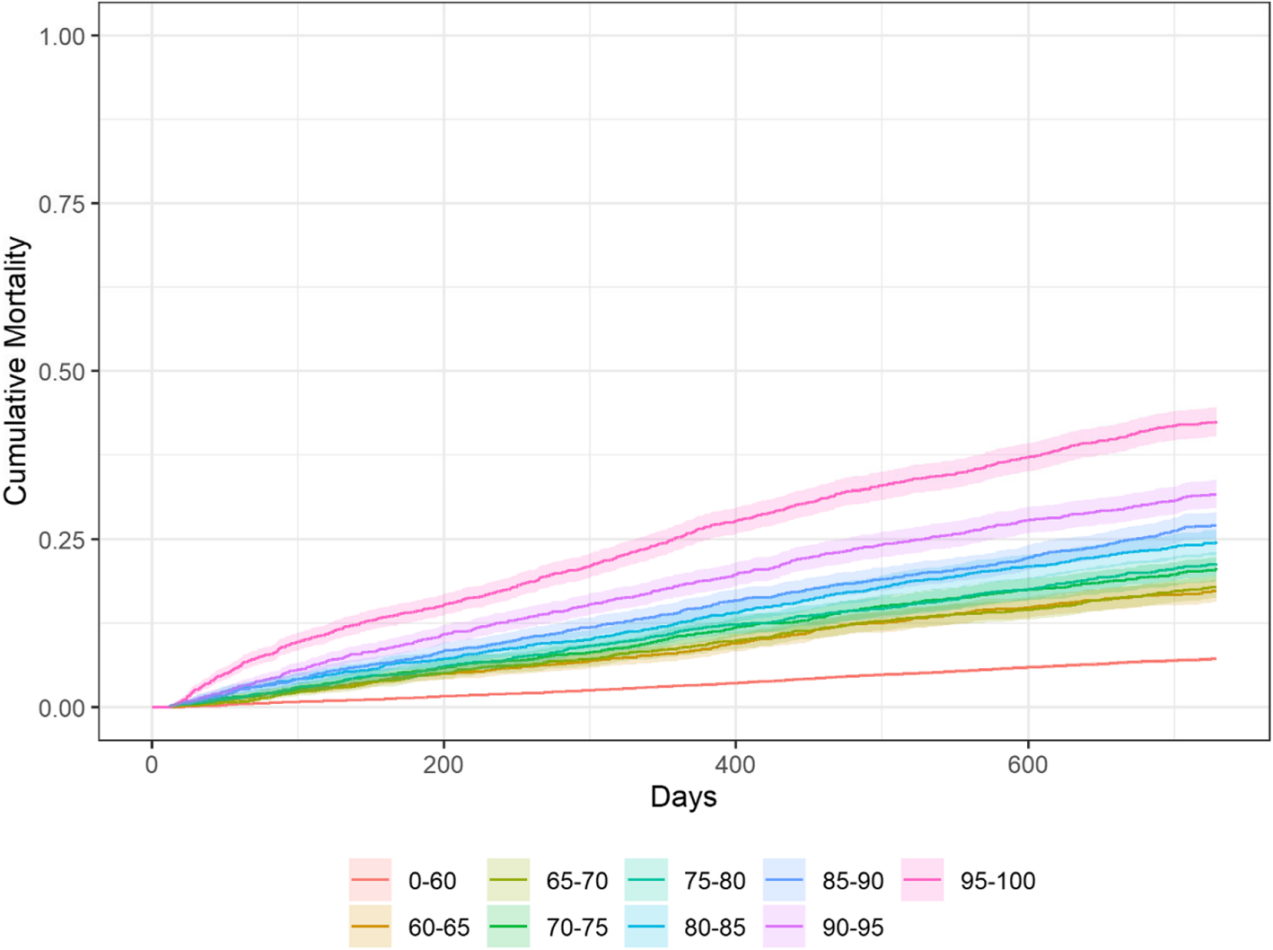
2-year cumulative mortality by percentiles of risk of hospital admission, year 2017–2018. Estimated using Kaplan–Meier

The cumulative mortality varied between 7 percent (percentiles < 60) and 43 percent (percentiles 95–100) with a follow-up time of two years. The confidence intervals overlapped between adjacent risk groups from percentiles 60–65 up to percentiles 85–90 and were thereafter separated (percentiles 90–95 and 95–100), see Table 3. Data on mortality are also presented in Fig. 2. There was no significant difference between men and women when divided into risk groups, based on overlapping confidence intervals.

**Table 3:** Cumulative mortality by percentiles of risk of hospital admission, year 2017–2018

Percentiles of risk of hospital admission	Number of individuals	Number of deaths	2-year cumulative mortality^a^, % (95% CI)	Hazard ratios^b^ (95% CI)
0–60	24 386	1 768	7 (7–8)	-
60–65	2 017	350	17 (16–19)	2.5 (2.3–2.9)
65–70	2 030	364	18 (16–20)	2.6 (2.4–2.9)
70–75	2 036	421	21 (19–22)	3.1 (2.8–3.4)
75–80	2 037	436	21 (20–23)	3.2 (2.9–3.6)
80–85	2 033	501	25 (23–27)	3.8 (3.4–4.2)
85–90	2 019	547	27 (25–29)	4.2 (3.8–4.6)
90–95	2 031	644	32 (30–34)	5.1 (4.7–5.6)
95–100	2 029	863	43 (40–45)	7.5 (6.9–8.2)
Total	40 618	5 894	15 (14–15)	-

*Abbreviations*: *CI* confidence interval

^a^Estimated using Kaplan–Meier

^b^Estimated using Cox-regression

**Fig. 3:**
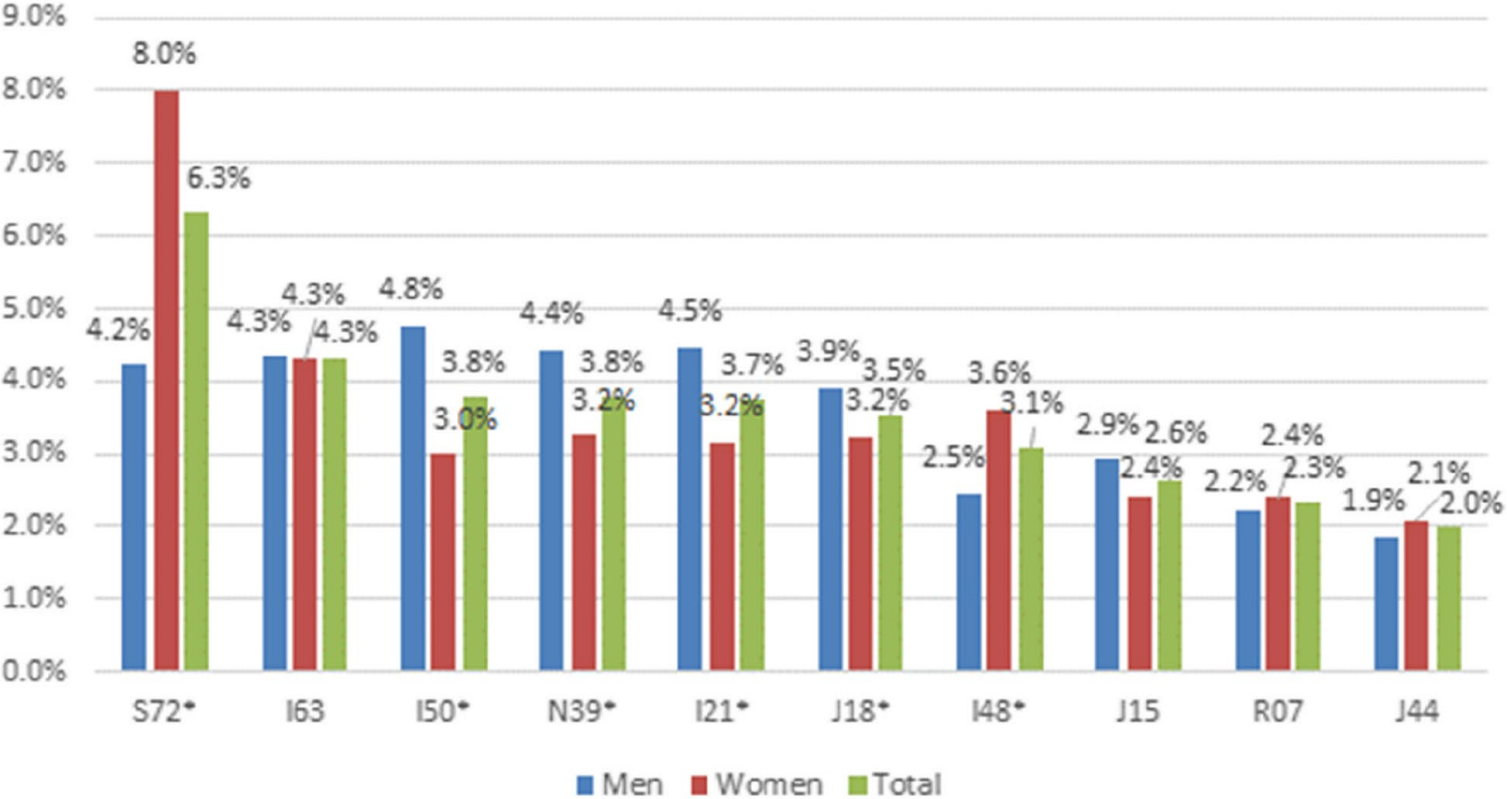
Distribution of top 10 primary discharge diagnoses for men and women, year 2017–2018. Presented according to ICD-10. S72 Fracture of femur, I63 Cerebral infarction, I50 Heart failure, N39 Other disorders of urinary system, I21 Acute myocardial infarction, J18 Pneumonia, organism unspecified, I48 Atrial fibrillation and flutter, J15 Bacterial pneumonia, not elsewhere classified, R07 Pain in throat and chest, J44 Other chronic obstructive pulmonary disease. *Statistical significance (Chi-2 test, *p* < 0.05) between men and women

In total there were 476 different primary discharge ICD-10 diagnoses after one year and 561 after two years. Ten ICD-10 diagnoses independently constituted 2.0 percent or more of all hospitalisation episodes. The diagnoses are listed in Fig. 3. When analysing primary discharge diagnosis according to ICD-10 blocks, there were significant differences between the groups (percentiles < 60, 60–85, 85–90, 90–95, and 95–100). However, the differences in percentage units were small and of unclear clinical relevance, and therefore only results divided into three risk groups are presented. The most common reason for hospitalisation according to ICD-10 blocks, divided into three risk groups are listed in Table 4. *Other forms of heart disease* (I30-I52) was the most common reason for hospitalisation, both in the study population in total and in all the risk groups. The second most frequent reason for all people, 75 years or older, and in the highest risk groups (percentiles 85–100) was *influenza and pneumonia* (J09-J18). *Influenza and pneumonia* was a frequent reason for hospitalisation in the lowest two risk groups (percentiles < 60 and 60–85) as well, but *injuries to the hip and thigh* (S70-S79) was more frequent.

**Table 4:** Distribution of primary discharge diagnosis according to ICD-10 block by risk of hospital admission

ICD-10 block	0–60	60–85	85–100	Total
I30-I52 Other forms of heart disease (%)	7.1^a^	9.1	11.0^b^	8.7
J09-J18 Influenza and pneumonia (%)	6.4^a^	8.2	9.5^b^	7.7
S70-S79 Injuries to the hip and thigh (%)	7.0	8.3^b^	5.2^a^	7.0
I20-I25 Ischaemic heart diseases (%)	6.4^b^	5.4	4.4^a^	5.6
I60-I69 Cerebrovascular diseases (%)	6.5^b^	5.1	3.8^a^	5.4
R00-R09 Symptoms and signs involving the circulatory and respiratory systems (%)	4.1	3.7	5.0^b^	4.2
N30-N39 Other diseases of urinary system (%)	3.9	3.8	4.2	4.0
R50-R69 General symptoms and signs (%)	3.6	3.3	3.3	3.4
K55-K64 Other diseases of intestines (%)	2.4	2.8	3.3	2.7
R40-R46 Symptoms and signs involving cognition, perception, emotional state and behaviour (%)	2.6	2.1	2.0	2.3
A30-A49 Other bacterial diseases (%)	1.7^a^	2.4	3.1^b^	2.3
J40-J47 Chronic lower respiratory diseases (%)	1.3^a^	2.3	4.0^b^	2.3
C00-C75 Malignant neoplasms, stated or presumed to be primary, of specified sites, except of lymphoid, haematopoietic and related tissue (%)	2.0	1.8	2.4	2.1
K80-K87 Disorders of gallbladder, biliary tract and pancreas (%)	2.4^b^	1.7	1.6	2.0
Other diagnoses (%)	42.5	39.9	38.8^a^	40.3
Total (%)	100	100	100	100
Number of individuals ≥ 1 hospital admissions	5 858	4 097	3 536	13 491

The most frequent ICD-10 diagnoses in each ICD-10 block were heart failure (I50), pneumonia, organism unspecified (J18), fracture of femur (S72), acute myocardial infarction (I21), cerebral infarction (I63), pain in throat and chest (R07), other disorders of urinary system (N39), syncope and collapse (R55), paralytic ileus and intestinal obstruction without hernia (K56), dizziness and giddiness (R42), erysipelas (A46), other chronic obstructive pulmonary disease (J44) malignant neoplasm of prostate (C61) and cholelithiasis (K80)

^a^Significantly lower than expected

^b^Significantly higher than expected

Among the ICD-10 diagnoses for the study population in total, *fracture of femur* (S72) was the most frequent reason for hospitalisation (6.3%), *cerebral infarction* (I63) the second (4.3%), and *heart failure* (I50) the third (3.8%) most frequent reason. For women, 75 years or older, *fracture of femur* was the most frequent discharge diagnosis (8.0%), *cerebral infarctio*n the second (4.3%), and *atrial fibrillation and flutter* (I48) the third most frequent (3.6%). For men, 75 years or older, the most frequent discharge diagnosis was *heart failure* (4.8%), the second *acute myocardial infarction* (I21) (4.5%), and the third *other disorders of urinary system* (N39) (4.4%).

For *other chronic obstructive pulmonary disease* (J44) 43 percent of the patients had the same discharge diagnosis the second episode, for the diagnosis *heart failure* 31 percent, and for *atrial fibrillation and flutter* 29 percent. These were the three diagnoses with the highest number of readmissions.

The five most frequent secondary discharge ICD-10 diagnoses were *essential hypertension* (I10) (12.1%), *atrial fibrillation and flutter* (I48) (6.1%), *chronic ischaemic heart disease* (I25) (4.4%), *personal history of medical treatment* (Z92) (4.2%), and *type 2 diabetes mellitus* (E11) (3.9%). For the highest risk group (percentiles 85–100) *essential hypertension* had a significant lower frequency than expected. For *atrial fibrillation and flutter*, *chronic ischaemic heart disease,* and *personal history of medical treatment* the highest risk group had a significant higher frequency than expected. For *type 2 diabetes mellitus* no significant difference from expected was found.

## Discussion

In the present study, the cumulative incidence of hospitalisation, the reasons for hospitalisation by diagnoses, and the cumulative mortality was explored to investigate how it varied between individuals, 75 years or older, at different levels of predicted risk of hospital admission.

The cumulative incidence of hospitalisation and the cumulative mortality increased with increasing risk of hospital admission, regardless of gender. From percentile 80 and upwards, the SHR for hospital admission ranged from 2.3 to 4.1 compared with individuals in the percentile < 60. The hazard ratios for mortality in the same percentiles ranged from 3.8 to 7.5.

It is well established that frail people have both higher mortality rate and higher risk of hospitalisation [6, 10, 39]. Individuals with high risk of hospital admission, according to the prediction model, also had higher risk for these outcomes. The increased risk for the population over percentile 80 was in the same range as frail individuals selected by the electronic frailty index developed by Clegg et al. [40]. However, in Clegg´s study the population was younger than in the present study, and the hazard ratios was calculated comparing frail individuals with fit individuals. The percentile < 60 has not been analysed in the present study, and it can therefore not be established whether these individuals were fit or not. Furthermore, frailty is more common among women than men [7, 19, 41], but the proportion of women decreased with increasing risk of hospitalisation in this study. Hence, even if frailty overlaps with risk of hospital admission, as expressed with the prediction model, they can be regarded as complimentary measures of risk, or vulnerability in an older person. We believe that the model could be used to identify vulnerable adults, 75 years or older, as a possible first step in screening for frailty.

As the frail population is heterogenous regarding care needs, the prediction model may have the advantage of finding individuals with the greatest needs. On the other hand, frail individuals that do not have frequent contacts with healthcare will not be identified by the model. Additional clinical methods will therefore always be of importance. To provide significant patient benefits, the use of the prediction model should be combined with a holistic clinical assessment of the patient. Future studies are needed to better understand the differences and similarities between frailty and predicted risk of hospital admission. It would also be valuable to investigate if the model can predict other negative outcomes, for example nursing home admission, or loss of functional ability.

Individuals with the highest risk of hospital admission were expected to have longer average length of stay during their first hospitalisation episode, reflecting complex medical needs and/or frailty. Several studies, but not all [42], have shown a correlation between frailty and average length of hospital stay [41, 43–45]. In the present study, the mean number of days for a hospitalisation episode did not differ between the risk-groups. However, the mean number of in-hospital days during the 2-year follow-up was considerably higher in the group with the highest risk of hospital admission.

The most common reason for hospitalisation were related to the circulatory and respiratory system, agreeing with previous studies [12, 13]. Women, 75 years or older, were hospitalised for femur fractures as the most frequent ICD-10 diagnosis, while for men, 75 years or older, heart failure was the main reason for hospitalisation.

The primary discharge diagnoses according to ICD-10 blocks are presented in three risk groups (percentiles < 60, 60–85, and 85–100) in this study. First, the ICD-10 blocks were analysed divided in five risk groups (percentiles < 60, 60–85, 85–90, 90–95, and 95–100). Even though several significant values could be found, the differences in percentage units were minimal. To make the result more manageable, the discharge diagnoses were presented in three risk groups, considering that the confidence intervals for the cumulative incidence of hospitalisation overlapped between adjacent risk groups between percentile 60 to 85. With this taken into consideration, the population, 75 years or older, could be divided into three groups (percentile < 60, 60–85, and 85–100). Even when presented in three risk-groups the differences were small and of unclear clinical relevance.

Pneumonia, chronic obstructive pulmonary disease (COPD), and heart failure are regarded as diagnoses where hospital admissions can be prevented if the patient is given optimal care in primary care and municipality [46–48]. These diagnoses were significantly more common in the highest risk-group (percentiles 85–100) in this study. COPD and heart failure were also the most frequent reasons for readmission with the same diagnosis as in the first hospitalisation episode. For women, 75 years or older, femur fracture was the most frequent discharge diagnosis. Thus, targeting risk for falls and pneumonia, as well as suboptimal treatment of COPD and heart failure could be part of an intervention to lower the rate of hospitalisation in a high-risk group. However, the difference in percent units was small, indicating that interventions to reduce hospitalisations should be personalised.

One strength of the study is that all individuals, 75 years or older, who lived in the county of Östergötland during the study period were included in the analysis. All hospitalisation episodes were included, regardless of where they occurred in Sweden. The registry used in the present study has almost non-existent dropouts and very little (negligible) missing data. There is, however, a small risk that some individuals moved from the region, which could impact the analyses of the hospitalisation, discharge diagnoses, and cumulative mortality.

Sweden has a low number of hospital beds per 1 000 people (2.1) compared with the mean for the European union (4.6) and even compared with the world in total (2.9) [49]. The average length of hospital stay in Sweden 2018 was 5.5 days, the fourth shortest in the European Union [50]. The relatively low number of hospital beds and short length of stay likely affect the average length of stay, the cumulative incidence of hospitalisation, and which diagnoses caused the hospitalisation. This makes it necessary to externally validate the model in other countries to be able to draw conclusions on the prediction model´s performance in other healthcare systems and generalisation of results. Further, discrepancies in coding of ICD-10 diagnoses between healthcare units and changes in the coding practice may impact the internal validity of the model. In order to avoid misclassification of individuals into high-risk or low-risk, it is of importance to combine the prediction model with a clinical assessment.

## Conclusion

Individuals, 75 years or older, identified by the prediction model to have high risk of hospital admission were demonstrated to have considerably higher cumulative incidence of hospitalisation, as well as mortality than individuals with lower risk. The results support the use of the prediction model to direct interventions towards groups in a population, 75 years or older, with the highest risk scores, and thus, likely the greatest care needs. In order to work resource-efficiently and reduce the risk of inpatient care, it is important to proactively identify these adults at high risk. The population selected by the prediction model partly overlap the population with frailty, but more studies are needed to describe this relation in more detail.

Heart failure, COPD, and pneumonia were significantly more frequent reason for hospitalisation for the individuals in the highest risk group. The women, 75 years or older, had fractures of the femur, and the men, 75 years or older, had heart failure as the most frequent discharge diagnosis. There were only small differences in discharge diagnoses between the risk groups, indicating that care interventions to reduce hospitalisations should be personalised and holistic, rather than disease-specific for people, 75 years or older, at high risk.

## Data Availability

The dataset used and/or analysed during the current study is available from the corresponding author on reasonable request.

## Abbreviations

CDW: Care Data Warehouse
CGA: Comprehensive Geriatric assessment
CI: Confidence interval
COPD: Chronic obstructive pulmonary disease
ER: Emergency room
ICD-10: International Classification of Diseases 10th Revision
SD: Standard deviation
SHR: Subdistribution hazard ratio
